# Minimally invasive mitral valve repair via right mini-thoracotomy in patient with myelodysplastic syndrome

**DOI:** 10.1186/s13019-018-0730-9

**Published:** 2018-05-18

**Authors:** Takura Taguchi, Hiroyuki Nishi, Kimihiro Kurose, Kohei Horikawa, Go Kanazawa, Toshiki Takahashi

**Affiliations:** Department of Cardiovascular Surgery, Osaka Police Hospital, 10-31, Kitayama-cho, Tennoji-ku, Osaka, 543-0035 Japan

**Keywords:** Myelodysplastic syndrome, Minimally invasive mitral valve repair, Right mini-thoracotomy

## Abstract

**Background:**

Cardiac surgery for myelodysplastic syndrome (MDS) patients is challenging because anemia and neutropenia develop as a result of the syndrome, leading to infection and bleeding tendency during surgery. We report the case of minimally invasive mitral valve repair via a right mini-thoracotomy and perioperative use of granulocyte colony-stimulating factor (G-CSF) in a patient with MDS.

**Case presentation:**

A 77-year-old man with myelodysplastic syndrome (MDS) was referred for surgical treatment for mitral valve regurgitation and underwent a minimally invasive mitral valve repair via a right mini-thoracotomy (MICS mitral procedure). On admission, laboratory results showed a leukocyte count of 1500/μL and neutrophils at 190/μL. Prior to surgery, a subcutaneous injection of granulocyte colony-stimulating factor (G-CSF) was given, based on a diagnosis of MDS by a hematologist. The MICS-mitral procedure using artificial chordae and an annular ring prosthesis was completed without requiring re-exploration for bleeding. Postoperatively, a G-CSF injection was administered and transfusion was required. There was no infection complication and the postoperative course was uneventful.

**Conclusion:**

A MICS-mitral procedure may be an effective option for MR patients with MDS who require a mitral valve repair to avoid postoperative infection and reduce the incidence of perioperative transfusion.

## Background

Myelodysplastic syndrome (MDS) is a set of oligoclonal disorders of hematopoietic stem cells characterized by ineffective hematopoiesis, which is clinically manifested as anemia, neutropenia, or thrombocytopenia of varying severity [1, 2]. Cardiac surgery for MDS patients is challenging because anemia and neutropenia develop as a result of the syndrome, leading to infection and bleeding tendency during surgery. Since extracorporeal circulation (ECC) increases the risk of bleeding, MDS patients undergoing cardiac surgery are more vulnerable due to a tendency for coagulopathy and infection [3]. Minimally invasive cardiac surgery (MICS) via a right thoracotomy is known to have a lower risk of postoperative bleeding and infection [4], and avoidance of a median sternotomy may be beneficial for patients with MDS. Furthermore, appropriate perioperative management is a key to success for patients with this uncommon disease. Herein, we report the case of minimally invasive mitral valve repair via a right mini-thoracotomy (MICS-mitral procedure) and perioperative use of granulocyte colony-stimulating factor (G-CSF) in a patient with MDS.

## Case presentation

A 77-year-old man was referred for surgical treatment for mitral valve regurgitation. There was a history of reflux esophagitis and his chief complaint was dyspnea on exertion during the previous 2 months. A physical examination revealed a pansystolic murmur at the apex. Electrocardiography showed a sinus rhythm and left ventricular hypertrophy, while echocardiography revealed severe mitral regurgitation due to A2-A3 prolapse with a dilated left ventricle (end-diastolic dimension/end-systolic dimension = 56/35 mm) and preserved left ventricular ejection fraction (EF = 65%). A coronary angiogram was normal.

Upon admission, laboratory test results showed the following: leukocytes 1500/μL, neutrophils 190/μL, hemoglobin 11.3 g/dL, and platelets 106 × 10^3^/μL. The patient was diagnosed with MDS by a hematologist, who advised us to administer G-CSF until the neutrophil count became greater than 1000/μL. No transfusion of packed red blood cells (RBCs) or platelet concentration (PC) was required.

**Fig. 1:**
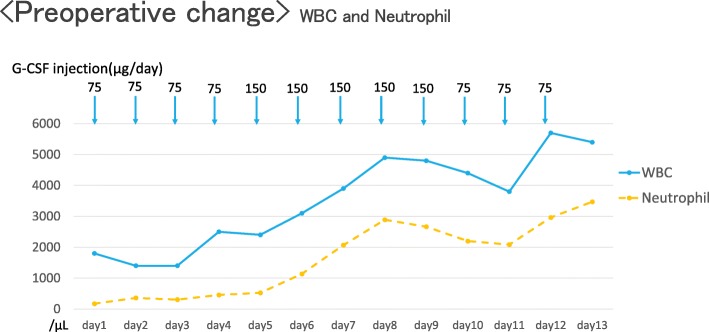
Preoperative time course of changes in white blood cells, and neutrophilsWBC: white blood cell, G-CSF: granulocyte colony-stimulating factor

A subcutaneous injection of G-CSF was given 12 days prior to surgery and the neutrophil count (3470/μL) gradually improved (Fig. 1). The patient underwent a MICS-mitral procedure through the fourth intercostal space with moderate hypothermia. Cardiopulmonary bypass (CPB) established via the right femoral artery and vein, along with antegrade and retrograde cardioplegia. The mitral valve was exposed through a left atriotomy, and torn chordae and prolapse of the A3 lesion were demonstrated. Two pairs of artificial chordae were implanted into posterior papillary muscle tissue and used for the anterior mitral leaflet of A3. An annular ring prosthesis (3D Memo Rechord #30) was used to fix the mitral annulus. During the operation, activated coagulation time was maintained at 349–612 s, and 2 units of packed RBCs, 4 units of fresh frozen plasma, and 40 units of PC were transfused in the operating room. Operation, CPB, and aortic cross-clamping times were 351, 209, and 143 min, respectively.

**Fig. 2:**
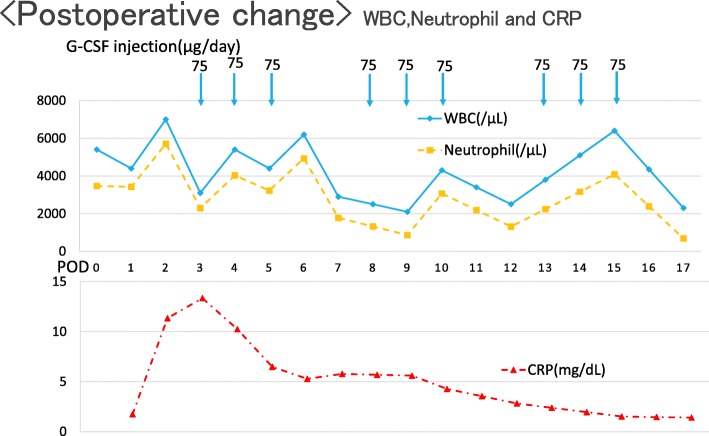
Postoperative time course of changes in white blood cells, neutrophils, and C-reactive protein. WBC: white blood cell, CRP: C-reactive protein, G-CSF: granulocyte colony-stimulating factor

The postoperative course was uneventful. There was no bleeding tendency and re-exploration for bleeding was not required. The patient was stable and was successfully extubated 37 h post surgery. Postoperatively, an injection of G-CSF, along with antibiotic prophylaxis of cefazolin (CEZ) and piperacillin (PIPC) were administered. The neutrophil count remained between 865 and 5712/μL (Fig. 2). Postoperative transfusions of 2 units of RBC and 40 units of PC were required. There was no complication with infection, while postoperative echocardiography revealed trivial mitral regurgitation with a normal ejection fraction. The patient was discharged on postoperative day 23 after confirming no sign of infection and that neutrophil numbers was within a normal range.

## Discussion

MDS is a heterogeneous group of blood diseases that usually presented as refractory anemia accompanied by various degrees of granulocytopenia and thrombocytopenia, with an approximately 25% risk of progression toward acute myeloid leukemia [5, 6]. The disease is caused by chronic and progressive blood dyscrasia, thus it often leads to an increased risk of infection and bleeding [1, 2]. As a result, the risk of mortality and morbidity is increased in the cases of patients with MDS undergoing major surgery. Cardiac surgery for MDS patients is rare, with only 13 cases reported thus far (Table 1). Most of those were performed due coronary artery bypass grafting [7–9] or aortic valve replacement [10–14] through a median sternotomy. Due to the variety of disease presentations, management and outcomes in previous reports differ. Because cardiac surgery requiring ECC itself results in more frequent complications, including infection and bleeding, as compared to that without ECC, comprehensive management to avoid complications is mandatory.

**Table 1:** Summary of open heart surgery procedures in patients with myelodysplastic syndrome

		Preoperative				Perioperative		
Author (year)	Age Sex	WBC (/μL)	Hb (g/dL)	Plt (/μL)	Surgery	G-CSF	Transfusion	Complication
Yamagish [7] 1996	61 M	1100	–	3000	CABG	+	+	–
Okamura [17] 1999	56 F	4400	11.3	130,000	MVR	–	+	–
Miyagi [8] 2001	79 M	1850	9.8	48,000	CABG	+	+	–
Yamashiro [18] 2002	68 M	3800	9.8	19,000	MVR	–	+	–
Kurisu [10] 2003	60 M	468 (neu)*	9.8	27,000	AVR	+	+	–
Hasegawa [19] 2003	70 M	1200	4.7	124,000	AVR Asc.Ao GR	+	+	Tamponade
Daitoku [11] 2007	73 F	1860	7.5	15,000	AVR	–	+	–
Tanaka [12] 2008	78 M	1600	5.6	–	AVR	+	+	–
Cavolli [9] 2010	70 M	1600	6.2	–	CABG	+	+	–
Okonogi [13] 2010	73 F	3700	10.1	34,000	AVR	–	+	–
Minami [20] 2011	73 F	3700	8.4	43,000	AVR Asc.Ao GR	+	+	SAH
Omoto [16] 2011	67 F	4600	11.9	307,000	MVP MAZE	–	+	Mediastinitis
Gangwani [14] 2014	66 M	3100	7.7	127,000	AVR	–	+	–
Present Case	77 M	1500	11.3	106,000	MVP	+	+	–

*CABG* coronary artery bypass grafting

*AVR* aortic valve replacement

*MVR* mitral valve replacement

*MVP* mitral valve plasty

*Asc.Ao GR* ascending aortic graft replacement

*SAH* subarachnoid hemorrhage

A right-mini thoracotomy approach for patients undergoing mitral valve surgery was introduced in the 1990s continues to undergo development, with MICS-mitral now been a popular treatment choice for mitral regurgitation [15]. Previous studies have found a tendency for a reduction in need for transfusion and a sternal infection can be avoided in patients who undergo a MICS-mitral procedure as compared to those undergoing mitral valve repair with a conventional full sternotomy [4]. Such reduced incidence of transfusion and no risk of sternum infection are major benefits. Furthermore, a mitral valve repair may be a preferable option as compared to mitral valve replacement in patients with MDS, because of the potential risks of prosthetic valve endocarditis and severe hemorrhage associated with Coumadin therapy. Therefore, we considered that a MICS-mitral procedure was suitable in the present case and, to the best of our knowledge, this is the first report of that procedure in a patient with MDS.

Coagulation disorders, with or without thrombocytopenia, are common in patients with MDS [1], as is platelet dysfunction, even in those with a normal platelet count. Cardiopulmonary bypass during cardiac surgery is itself associated with changes in the coagulation system, mainly due to the contact of blood and its components with the ECC circuit. Therefore, it is important to appropriately manage bleeding in MDS patients. We chose an MICS-mitral procedure and avoided a median sternotomy, which causes bleeding from bone marrow, thus relatively few RBC packs and a low amount of fresh frozen plasma were required for the present case. On the other hand, a large amount of PC was needed, as noted in previous cases, because our patient had a more severe bleeding tendency than expected.

Another important issue is prevention of infection. Patients with MDS have increased risk of infection during and after surgery. G-CSF is used for treatment of patients with MDS to increase neutrophil count, and may reduce the number of affected patients or severity of infection [2]. Although no transfusion of RBCs or platelets were needed preoperatively in the present case, a preoperative G-CSF injection was administered to keep the neutrophil count above 1000/μL, which was done in cooperation with a hematologist. Also, postoperative G-CSF was required to maintain the neutrophil count at an appropriate level. As a result, there was no serious perioperative infection. Comprehensive prophylactic management including appropriate use of antibiotics and preoperative preparation also contributed to our good results. A high risk of wound infection after open heart surgery in patients with hematologic malignancies has been reported [3], including a case of mediastinitis following mitral valve repair in a patient with MDS [16]. In the present patient, we were not concerned about a sternum infection due to use of the MICS-mitral procedure, which might be another great advantage for patients with MDS.

## Conclusion

Prevention of infection and appropriate management of bleeding tendency by careful planning and cooperation among the attending hematologist, anesthetist, and surgeon are mandatory for MDS patients who undergo open heart surgery. A MICS-mitral procedure may be an effective option for MR patients with MDS who require a mitral valve repair to avoid postoperative infection and reduce the incidence of perioperative transfusion.

## Abbreviations

CEZ: Cefazolin
CPB: Cardiopulmonary bypass
CRP: C-reactive protein
ECC: Extracorporeal circulation
G-CSF: Granulocyte colony-stimulating factor
MDS: Myelodysplastic syndrome
MICS: Minimally invasive cardiac surgery
PC: Platelet concentration
PIPC: Piperacillin
RBC: Red blood cell
WBC: White blood cell
